# Bis(2,2′-bipyridine-κ^2^ *N*,*N*′)dichloridocadmium(II)

**DOI:** 10.1107/S1600536810049251

**Published:** 2010-11-30

**Authors:** Shan Gao, Seik Weng Ng

**Affiliations:** a College of Chemistry and Materials Science, Heilongjiang University, Harbin 150080, People’s Republic of China; b Department of Chemistry, University of Malaya, 50603 Kuala Lumpur, Malaysia

## Abstract

The Cd^II^ atom in the title compound, [CdCl_2_(C_10_H_8_N_2_)_2_] exists in a distorted octa­hedral geometry [N—Cd—N = 148.29 (17)°]; the Cl atoms are *cis* with respect to each other.

## Related literature

For polymeric dichlorido(2,2′-bipyridine)­cadmium, see: Zhou *et al.* (2003 ▶).
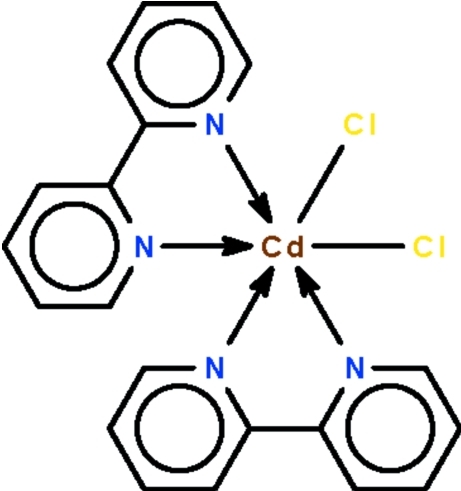


## Experimental

### 

#### Crystal data

[CdCl_2_(C_10_H_8_N_2_)_2_]
*M* *_r_* = 495.67
Monoclinic, 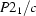

*a* = 8.7477 (2) Å
*b* = 14.3541 (5) Å
*c* = 15.8723 (5) Å
β = 98.775 (1)°
*V* = 1969.68 (10) Å^3^
*Z* = 4
Mo *K*α radiation
μ = 1.39 mm^−1^
*T* = 293 K
0.18 × 0.15 × 0.12 mm

#### Data collection

Rigaku R-AXIS RAPID diffractometer
Absorption correction: multi-scan (*ABSCOR*; Higashi, 1995 ▶) *T*_min_ = 0.788, *T*_max_ = 0.851
31202 measured reflections
4497 independent reflections
3047 reflections with *I* > 2σ(*I*)
*R*_int_ = 0.056

#### Refinement

*R*[*F* ^2^ > 2σ(*F* ^2^)] = 0.053
*wR*(*F* ^2^) = 0.186
*S* = 1.17
4497 reflections
245 parameters
H-atom parameters constrained
Δρ_max_ = 1.61 e Å^−3^
Δρ_min_ = −1.04 e Å^−3^

### 

Data collection: *RAPID-AUTO* (Rigaku, 1998 ▶); cell refinement: *RAPID-AUTO*; data reduction: *CrystalStructure* (Rigaku/MSC, 2002 ▶); program(s) used to solve structure: *SHELXS97* (Sheldrick, 2008 ▶); program(s) used to refine structure: *SHELXL97* (Sheldrick, 2008 ▶); molecular graphics: *X-SEED* (Barbour, 2001 ▶); software used to prepare material for publication: *publCIF* (Westrip, 2010 ▶).

## Supplementary Material

Crystal structure: contains datablocks global, I. DOI: 10.1107/S1600536810049251/jh2233sup1.cif

Structure factors: contains datablocks I. DOI: 10.1107/S1600536810049251/jh2233Isup2.hkl

Additional supplementary materials:  crystallographic information; 3D view; checkCIF report

## Figures and Tables

**Fig. 1.**
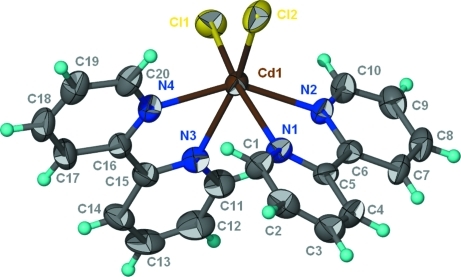
Thermal ellipsoid plot (Barbour, 2001) of CdCl2(C10H8N2)2 at the 50% probability level; hydrogen atoms are drawn as spheres of arbitrary radius.

## supplementary crystallographic information

### Comment

The hydrothermal reaction of cadmium chloride and 2,2'-bipyridine yields the 1:1 adduct, which exists as a chlorine-bridged chain polymer. The cadmium atom exists in an octahedral geometry (Zhou *et al.*, 2003). In the present 1:2 adduct (Scheme I, Fig. 1), the geometry is also an octahedron but the molecule exists as a discrete entity, without any bridging.

### Experimental

Cadmium chloride (0.1 mmol), 2,2'-bipyridine (0.1 mmol) and benzoic acid (0.2 mmol) were dissolved in a water-ethanol-DMF mixture (15 ml). The solution was heated in a 25 ml, Teflon-lined, stainless-steel bomb at 383 K for 3 days. The cool solution was filtered and the solvent allowed to evaporate. Colorless crystals separated from solution after a few days.

### Refinement

Hydrogen atoms were placed in calculated positions (C–H 0.93 Å) and were included in the refinement in the riding model approximation, with *U*(H) set to 1.2*U*_eq_(C).

### Crystal data

**Table:**

[CdCl_2_(C_10_H_8_N_2_)_2_]	*F*(000) = 984
*M**_r_* = 495.67	*D*_x_ = 1.671 Mg m^−^^3^
Monoclinic, *P*2_1_/*c*	Mo *K*α radiation, λ = 0.71073 Å
Hall symbol: -P 2ybc	Cell parameters from 18413 reflections
*a* = 8.7477 (2) Å	θ = 3.1–27.4°
*b* = 14.3541 (5) Å	µ = 1.39 mm^−^^1^
*c* = 15.8723 (5) Å	*T* = 293 K
β = 98.775 (1)°	Block, colorless
*V* = 1969.68 (10) Å^3^	0.18 × 0.15 × 0.12 mm
*Z* = 4	

### Data collection

**Table:**

Rigaku R-AXIS RAPID diffractometer	4497 independent reflections
Radiation source: fine-focus sealed tube	3047 reflections with *I* > 2σ(*I*)
graphite	*R*_int_ = 0.056
Detector resolution: 10.000 pixels mm^-1^	θ_max_ = 27.4°, θ_min_ = 3.1°
ω scans	*h* = −11→10
Absorption correction: multi-scan (*ABSCOR*; Higashi, 1995)	*k* = −18→18
*T*_min_ = 0.788, *T*_max_ = 0.851	*l* = −20→20
31202 measured reflections	

### Refinement

**Table:**

Refinement on *F*^2^	Secondary atom site location: difference Fourier map
Least-squares matrix: full	Hydrogen site location: inferred from neighbouring sites
*R*[*F*^2^ > 2σ(*F*^2^)] = 0.053	H-atom parameters constrained
*wR*(*F*^2^) = 0.186	*w* = 1/[σ^2^(*F*_o_^2^) + (0.1002*P*)^2^ + 1.1952*P*] where *P* = (*F*_o_^2^ + 2*F*_c_^2^)/3
*S* = 1.17	(Δ/σ)_max_ = 0.001
4497 reflections	Δρ_max_ = 1.61 e Å^−^^3^
245 parameters	Δρ_min_ = −1.04 e Å^−^^3^
0 restraints	Extinction correction: *SHELXL97* (Sheldrick, 2008), Fc^*^=kFc[1+0.001xFc^2^λ^3^/sin(2θ)]^-1/4^
Primary atom site location: structure-invariant direct methods	Extinction coefficient: 0.0073 (12)

### Fractional atomic coordinates and isotropic or equivalent isotropic displacement parameters (Å^2^)

**Table:**

	*x*	*y*	*z*	*U*_iso_*/*U*_eq_	
Cd1	0.69488 (4)	0.77175 (3)	0.53520 (3)	0.0434 (2)	
Cl1	0.4692 (2)	0.77680 (12)	0.41696 (13)	0.0690 (5)	
Cl2	0.6158 (2)	0.88210 (14)	0.64297 (14)	0.0766 (6)	
N1	0.9503 (6)	0.7787 (3)	0.6172 (3)	0.0471 (12)	
N2	0.8703 (5)	0.8562 (3)	0.4634 (3)	0.0445 (11)	
N3	0.7797 (6)	0.6284 (4)	0.4710 (3)	0.0504 (12)	
N4	0.6420 (5)	0.6347 (4)	0.6118 (3)	0.0492 (12)	
C1	0.9828 (8)	0.7475 (5)	0.6967 (4)	0.0565 (16)	
H1	0.9028	0.7215	0.7213	0.068*	
C2	1.1294 (9)	0.7513 (5)	0.7455 (5)	0.0645 (19)	
H2	1.1465	0.7301	0.8015	0.077*	
C3	1.2469 (9)	0.7873 (5)	0.7080 (5)	0.068 (2)	
H3	1.3470	0.7892	0.7379	0.081*	
C4	1.2168 (7)	0.8207 (5)	0.6260 (5)	0.0622 (17)	
H4	1.2961	0.8455	0.6001	0.075*	
C5	1.0667 (6)	0.8170 (4)	0.5820 (4)	0.0463 (13)	
C6	1.0215 (6)	0.8603 (4)	0.4967 (4)	0.0455 (13)	
C7	1.1268 (7)	0.9075 (5)	0.4553 (4)	0.0576 (16)	
H7	1.2309	0.9093	0.4787	0.069*	
C8	1.0756 (8)	0.9521 (5)	0.3786 (4)	0.0661 (19)	
H8	1.1450	0.9838	0.3500	0.079*	
C9	0.9192 (9)	0.9489 (5)	0.3451 (4)	0.0652 (18)	
H9	0.8808	0.9789	0.2944	0.078*	
C10	0.8230 (8)	0.8991 (5)	0.3903 (4)	0.0548 (15)	
H10	0.7185	0.8956	0.3678	0.066*	
C11	0.8480 (8)	0.6289 (5)	0.4022 (4)	0.0635 (18)	
H11	0.8691	0.6861	0.3790	0.076*	
C12	0.8900 (9)	0.5483 (7)	0.3628 (5)	0.080 (2)	
H12	0.9375	0.5513	0.3143	0.096*	
C13	0.8595 (9)	0.4645 (6)	0.3976 (5)	0.079 (3)	
H13	0.8872	0.4092	0.3733	0.095*	
C14	0.7875 (8)	0.4625 (5)	0.4688 (5)	0.066 (2)	
H14	0.7663	0.4060	0.4931	0.080*	
C15	0.7461 (7)	0.5474 (4)	0.5046 (4)	0.0493 (14)	
C16	0.6667 (6)	0.5509 (4)	0.5800 (4)	0.0470 (14)	
C17	0.6114 (8)	0.4700 (5)	0.6166 (5)	0.0648 (18)	
H17	0.6267	0.4116	0.5939	0.078*	
C18	0.5352 (8)	0.4779 (6)	0.6858 (5)	0.074 (2)	
H18	0.4980	0.4251	0.7100	0.089*	
C19	0.5145 (8)	0.5644 (6)	0.7189 (5)	0.068 (2)	
H19	0.4647	0.5716	0.7662	0.081*	
C20	0.5707 (7)	0.6415 (5)	0.6792 (4)	0.0584 (16)	
H20	0.5573	0.7004	0.7014	0.070*	

### Atomic displacement parameters (Å^2^)

**Table:**

	*U*^11^	*U*^22^	*U*^33^	*U*^12^	*U*^13^	*U*^23^
Cd1	0.0380 (3)	0.0474 (3)	0.0449 (3)	−0.00430 (17)	0.00717 (18)	−0.00333 (18)
Cl1	0.0532 (9)	0.0678 (11)	0.0776 (12)	0.0016 (8)	−0.0165 (9)	−0.0071 (9)
Cl2	0.0647 (10)	0.0750 (12)	0.0984 (14)	−0.0204 (9)	0.0392 (10)	−0.0371 (11)
N1	0.041 (3)	0.056 (3)	0.042 (3)	−0.001 (2)	−0.001 (2)	0.001 (2)
N2	0.038 (2)	0.050 (3)	0.045 (3)	−0.008 (2)	0.0037 (19)	0.003 (2)
N3	0.051 (3)	0.055 (3)	0.045 (3)	0.002 (2)	0.008 (2)	−0.001 (2)
N4	0.047 (3)	0.051 (3)	0.050 (3)	−0.009 (2)	0.006 (2)	0.001 (2)
C1	0.044 (3)	0.071 (4)	0.053 (4)	−0.005 (3)	0.000 (3)	−0.001 (3)
C2	0.072 (5)	0.060 (4)	0.055 (4)	0.008 (3)	−0.011 (4)	−0.005 (3)
C3	0.055 (4)	0.064 (4)	0.078 (5)	0.002 (3)	−0.013 (4)	−0.003 (4)
C4	0.038 (3)	0.067 (4)	0.080 (5)	−0.012 (3)	0.001 (3)	−0.003 (4)
C5	0.036 (3)	0.046 (3)	0.057 (3)	0.004 (2)	0.005 (2)	−0.004 (3)
C6	0.043 (3)	0.049 (3)	0.047 (3)	0.001 (2)	0.015 (2)	−0.011 (3)
C7	0.050 (3)	0.059 (4)	0.067 (4)	−0.018 (3)	0.020 (3)	−0.001 (3)
C8	0.075 (5)	0.064 (4)	0.066 (4)	−0.021 (4)	0.032 (4)	−0.001 (3)
C9	0.083 (5)	0.061 (4)	0.053 (4)	−0.006 (4)	0.014 (3)	0.004 (3)
C10	0.058 (4)	0.061 (4)	0.044 (3)	−0.009 (3)	0.006 (3)	0.009 (3)
C11	0.071 (4)	0.066 (4)	0.059 (4)	0.005 (4)	0.024 (3)	−0.009 (3)
C12	0.078 (5)	0.092 (7)	0.070 (5)	0.013 (5)	0.016 (4)	−0.030 (5)
C13	0.072 (5)	0.080 (6)	0.083 (6)	0.024 (4)	0.003 (4)	−0.042 (5)
C14	0.066 (4)	0.047 (4)	0.080 (5)	0.008 (3)	−0.007 (4)	−0.014 (3)
C15	0.045 (3)	0.046 (3)	0.054 (3)	0.002 (3)	−0.003 (3)	−0.006 (3)
C16	0.039 (3)	0.045 (3)	0.052 (3)	−0.005 (2)	−0.007 (2)	0.005 (3)
C17	0.062 (4)	0.049 (4)	0.079 (5)	−0.011 (3)	−0.002 (4)	0.018 (3)
C18	0.057 (4)	0.076 (5)	0.085 (5)	−0.018 (4)	−0.001 (4)	0.037 (5)
C19	0.054 (4)	0.092 (6)	0.054 (4)	−0.015 (4)	−0.002 (3)	0.018 (4)
C20	0.054 (3)	0.075 (5)	0.049 (3)	−0.011 (3)	0.017 (3)	0.001 (3)

### Geometric parameters (Å, °)

**Table:**

Cd1—N2	2.378 (5)	C7—C8	1.388 (9)
Cd1—N4	2.394 (5)	C7—H7	0.9300
Cd1—N1	2.410 (5)	C8—C9	1.391 (10)
Cd1—N3	2.461 (5)	C8—H8	0.9300
Cd1—Cl2	2.5049 (18)	C9—C10	1.385 (9)
Cd1—Cl1	2.5087 (18)	C9—H9	0.9300
N1—C1	1.327 (8)	C10—H10	0.9300
N1—C5	1.351 (8)	C11—C12	1.392 (10)
N2—C10	1.323 (7)	C11—H11	0.9300
N2—C6	1.349 (7)	C12—C13	1.366 (13)
N3—C11	1.322 (8)	C12—H12	0.9300
N3—C15	1.330 (8)	C13—C14	1.375 (10)
N4—C20	1.322 (8)	C13—H13	0.9300
N4—C16	1.335 (8)	C14—C15	1.415 (9)
C1—C2	1.395 (10)	C14—H14	0.9300
C1—H1	0.9300	C15—C16	1.472 (10)
C2—C3	1.365 (11)	C16—C17	1.417 (9)
C2—H2	0.9300	C17—C18	1.374 (11)
C3—C4	1.375 (10)	C17—H17	0.9300
C3—H3	0.9300	C18—C19	1.370 (12)
C4—C5	1.391 (8)	C18—H18	0.9300
C4—H4	0.9300	C19—C20	1.399 (10)
C5—C6	1.487 (8)	C19—H19	0.9300
C6—C7	1.387 (8)	C20—H20	0.9300
			
N2—Cd1—N4	148.29 (17)	C7—C6—C5	122.2 (5)
N2—Cd1—N1	67.98 (16)	C6—C7—C8	119.5 (6)
N4—Cd1—N1	89.69 (16)	C6—C7—H7	120.2
N2—Cd1—N3	88.30 (17)	C8—C7—H7	120.2
N4—Cd1—N3	67.48 (19)	C7—C8—C9	119.1 (6)
N1—Cd1—N3	86.83 (16)	C7—C8—H8	120.5
N2—Cd1—Cl2	105.71 (13)	C9—C8—H8	120.5
N4—Cd1—Cl2	94.47 (13)	C10—C9—C8	117.4 (6)
N1—Cd1—Cl2	86.28 (13)	C10—C9—H9	121.3
N3—Cd1—Cl2	160.70 (13)	C8—C9—H9	121.3
N2—Cd1—Cl1	96.80 (12)	N2—C10—C9	124.1 (6)
N4—Cd1—Cl1	102.28 (12)	N2—C10—H10	117.9
N1—Cd1—Cl1	164.08 (14)	C9—C10—H10	117.9
N3—Cd1—Cl1	88.10 (12)	N3—C11—C12	123.4 (8)
Cl2—Cd1—Cl1	102.99 (7)	N3—C11—H11	118.3
C1—N1—C5	117.7 (5)	C12—C11—H11	118.3
C1—N1—Cd1	123.1 (4)	C13—C12—C11	118.0 (7)
C5—N1—Cd1	119.2 (4)	C13—C12—H12	121.0
C10—N2—C6	118.7 (5)	C11—C12—H12	121.0
C10—N2—Cd1	121.3 (4)	C12—C13—C14	119.5 (7)
C6—N2—Cd1	120.0 (4)	C12—C13—H13	120.3
C11—N3—C15	119.5 (6)	C14—C13—H13	120.3
C11—N3—Cd1	122.7 (5)	C13—C14—C15	119.4 (7)
C15—N3—Cd1	117.7 (4)	C13—C14—H14	120.3
C20—N4—C16	119.7 (6)	C15—C14—H14	120.3
C20—N4—Cd1	120.2 (5)	N3—C15—C14	120.3 (6)
C16—N4—Cd1	119.6 (4)	N3—C15—C16	117.2 (5)
N1—C1—C2	124.1 (7)	C14—C15—C16	122.5 (6)
N1—C1—H1	118.0	N4—C16—C17	119.9 (6)
C2—C1—H1	118.0	N4—C16—C15	117.4 (5)
C3—C2—C1	117.5 (7)	C17—C16—C15	122.6 (6)
C3—C2—H2	121.2	C18—C17—C16	119.9 (7)
C1—C2—H2	121.2	C18—C17—H17	120.1
C2—C3—C4	119.8 (7)	C16—C17—H17	120.1
C2—C3—H3	120.1	C19—C18—C17	119.3 (7)
C4—C3—H3	120.1	C19—C18—H18	120.3
C3—C4—C5	119.4 (6)	C17—C18—H18	120.3
C3—C4—H4	120.3	C18—C19—C20	117.9 (7)
C5—C4—H4	120.3	C18—C19—H19	121.0
N1—C5—C4	121.5 (6)	C20—C19—H19	121.0
N1—C5—C6	115.8 (5)	N4—C20—C19	123.2 (7)
C4—C5—C6	122.7 (6)	N4—C20—H20	118.4
N2—C6—C7	121.2 (6)	C19—C20—H20	118.4
N2—C6—C5	116.5 (5)		
			
N2—Cd1—N1—C1	173.2 (5)	Cd1—N1—C5—C4	−178.5 (5)
N4—Cd1—N1—C1	−29.9 (5)	C1—N1—C5—C6	−173.5 (5)
N3—Cd1—N1—C1	−97.4 (5)	Cd1—N1—C5—C6	5.6 (7)
Cl2—Cd1—N1—C1	64.6 (5)	C3—C4—C5—N1	−2.1 (10)
Cl1—Cd1—N1—C1	−169.0 (4)	C3—C4—C5—C6	173.6 (6)
N2—Cd1—N1—C5	−5.8 (4)	C10—N2—C6—C7	−0.7 (9)
N4—Cd1—N1—C5	151.1 (4)	Cd1—N2—C6—C7	178.7 (5)
N3—Cd1—N1—C5	83.6 (4)	C10—N2—C6—C5	175.5 (5)
Cl2—Cd1—N1—C5	−114.4 (4)	Cd1—N2—C6—C5	−5.0 (7)
Cl1—Cd1—N1—C5	12.0 (8)	N1—C5—C6—N2	−0.4 (8)
N4—Cd1—N2—C10	136.8 (5)	C4—C5—C6—N2	−176.3 (6)
N1—Cd1—N2—C10	−175.0 (5)	N1—C5—C6—C7	175.8 (6)
N3—Cd1—N2—C10	97.8 (5)	C4—C5—C6—C7	−0.1 (10)
Cl2—Cd1—N2—C10	−95.7 (5)	N2—C6—C7—C8	0.6 (10)
Cl1—Cd1—N2—C10	9.9 (5)	C5—C6—C7—C8	−175.4 (6)
N4—Cd1—N2—C6	−42.7 (6)	C6—C7—C8—C9	0.3 (11)
N1—Cd1—N2—C6	5.6 (4)	C7—C8—C9—C10	−1.2 (11)
N3—Cd1—N2—C6	−81.7 (4)	C6—N2—C10—C9	−0.2 (10)
Cl2—Cd1—N2—C6	84.9 (4)	Cd1—N2—C10—C9	−179.6 (5)
Cl1—Cd1—N2—C6	−169.5 (4)	C8—C9—C10—N2	1.1 (11)
N2—Cd1—N3—C11	−20.4 (5)	C15—N3—C11—C12	−1.1 (11)
N4—Cd1—N3—C11	−179.4 (5)	Cd1—N3—C11—C12	−176.8 (6)
N1—Cd1—N3—C11	−88.4 (5)	N3—C11—C12—C13	−0.4 (12)
Cl2—Cd1—N3—C11	−157.7 (4)	C11—C12—C13—C14	0.8 (12)
Cl1—Cd1—N3—C11	76.5 (5)	C12—C13—C14—C15	0.1 (11)
N2—Cd1—N3—C15	163.8 (4)	C11—N3—C15—C14	2.0 (9)
N4—Cd1—N3—C15	4.8 (4)	Cd1—N3—C15—C14	178.0 (4)
N1—Cd1—N3—C15	95.7 (4)	C11—N3—C15—C16	−178.6 (5)
Cl2—Cd1—N3—C15	26.5 (7)	Cd1—N3—C15—C16	−2.6 (7)
Cl1—Cd1—N3—C15	−99.4 (4)	C13—C14—C15—N3	−1.6 (10)
N2—Cd1—N4—C20	138.8 (4)	C13—C14—C15—C16	179.0 (6)
N1—Cd1—N4—C20	95.0 (5)	C20—N4—C16—C17	2.3 (9)
N3—Cd1—N4—C20	−178.3 (5)	Cd1—N4—C16—C17	−169.2 (4)
Cl2—Cd1—N4—C20	8.7 (5)	C20—N4—C16—C15	179.8 (5)
Cl1—Cd1—N4—C20	−95.6 (4)	Cd1—N4—C16—C15	8.3 (7)
N2—Cd1—N4—C16	−49.8 (6)	N3—C15—C16—N4	−3.6 (8)
N1—Cd1—N4—C16	−93.6 (4)	C14—C15—C16—N4	175.8 (5)
N3—Cd1—N4—C16	−6.9 (4)	N3—C15—C16—C17	173.8 (5)
Cl2—Cd1—N4—C16	−179.8 (4)	C14—C15—C16—C17	−6.8 (10)
Cl1—Cd1—N4—C16	75.9 (4)	N4—C16—C17—C18	−1.2 (9)
C5—N1—C1—C2	−0.5 (10)	C15—C16—C17—C18	−178.6 (6)
Cd1—N1—C1—C2	−179.6 (5)	C16—C17—C18—C19	−0.4 (10)
N1—C1—C2—C3	−1.7 (11)	C17—C18—C19—C20	1.0 (10)
C1—C2—C3—C4	2.1 (11)	C16—N4—C20—C19	−1.8 (10)
C2—C3—C4—C5	−0.3 (11)	Cd1—N4—C20—C19	169.6 (5)
C1—N1—C5—C4	2.5 (9)	C18—C19—C20—N4	0.1 (11)
